# Women’s barriers for contacting general practice when experiencing gynecological cancer symptoms: a population-based study

**DOI:** 10.1186/s12875-021-01518-5

**Published:** 2021-08-16

**Authors:** Kirubakaran Balasubramaniam, Sanne Rasmussen, Peter Fentz Haastrup, Kaspar Suadicani, Jens Søndergaard, Dorte Ejg Jarbøl

**Affiliations:** 1 Department of Public Health, Research Unit of General Practice, University of Southern Denmark, J. B. Winsløws Vej 9A, 5000 Odense C, Denmark; 2 University of Southern Denmark, Odense, Denmark

**Keywords:** Gynecological cancer, Symptoms, Healthcare seeking, Barriers, Socioeconomic status

## Abstract

**Background:**

A prerequisite for general practitioners (GPs) being able to refer patients with gynecological cancer alarm symptoms for further investigations is that individuals present the symptoms to the GP. Not all symptoms are presented to the GP, and knowledge of barriers for healthcare-seeking is sparse. The aim of this study was to analyze associations between age, socioeconomic status, and common barriers (“being too embarrassed”, “being too busy”, “worried about wasting the doctors time” and “worried what the GP might find”) towards GP contact with gynecological alarm symptoms.

**Methods:**

Nationwide population-based study in Denmark based on a random sample of 51 090 women aged 20 years or older. A web-based questionnaire regarding experience of four predefined alarm symptoms of gynecological cancer, decisions about contact to GPs, and barriers towards GP contact was distributed. Information about socioeconomic status was collected from Statistics Denmark.

**Results:**

A total of 26 466 women (54.5%) completed the questionnaire. The proportion of women with no contact to the GP varied between 64.6% and 78.1% for postmenopausal bleeding and pain during intercourse, respectively. Between 32.3% (bleeding during intercourse) and 45.3% (postmenopausal bleeding) of the women reported no barriers for GP contact. The proportions of reported barriers ranged from 7.5% for being too embarrassed (pelvic pain) to 26.8% for being too busy (bleeding during intercourse).

Women aged 40–59 years had lower odds of reporting “being too embarrassed” and “worried about wasting the GP´s time”, while women aged 60 + years of age had lower odds of reporting “being too busy” compared to the youngest age group.

Women in the highest income groups had lower odds of reporting “being too embarrassed” and “wasting the GP´s time” compared to those with a low income, while those with high educational level had lower odds of reporting “being too embarrassed” and “worried what the GP might find” compared to those with low educational level.

**Conclusions:**

More than half of the respondents with no contact to the GP, reported one or more barriers towards GP contact. Lower age and socioeconomic status were significantly associated with higher odds of reporting barriers. As this may explain the differences in healthcare seeking behavior, healthcare planners, policy makers and clinicians should be aware of these findings.

## Background

Stage at diagnosis is one of the most important prognostic factors for cancer survival, [1] and for most types of cancer including the gynecological cancers, prolonged diagnostic interval is strongly associated with more advanced stage at diagnosis [2]. Therefore, referral guidelines for general practice have been introduced to expedite the diagnostic process for different gynecological cancers. These guidelines suggest fast track referral of women with alarm symptoms of malignancy, e.g. postmenopausal bleeding or bleeding during intercourse [3, 4]. However, a prerequisite for referral is that the symptoms are presented to the general practitioner (GP) which is not always the case, and the literature suggests that especially women with gynecological malignancies have prolonged time from symptom presentation to GP contact [5]. Several studies have shown that the decision of healthcare seeking arises in a complex interplay of symptom characteristics and numerous socioeconomic and psychosocial factors related to the individual [6–10]. In a previous study, we found that socioeconomic characteristics such as increasing age, high educational level and immigrant status are associated with GP contact when experiencing gynecological alarm symptoms [11]. From studies of healthcare-seeking we know that different barriers exist for contacting the GP when experiencing an alarm symptom such as being embarrassed, worrying about wasting the GP’s time, being afraid of what the GP might find and being too busy to consult the GP [12, 13]. Differences in barriers for healthcare-seeking may vary between socioeconomic groups and could be a key to understand why socioeconomic factors are associated with GP contact when experiencing gynecological alarm symptoms [14].

Thus, the aim of this paper is to analyze associations between four common barriers towards contacting the GP regarding specific gynecological cancer alarm symptoms, age and socioeconomic status.

## Methods

We conducted a nationwide population-based cohort study. A sample of 100 000 individuals aged 20 years or older was randomly selected from the general population using the Danish Civil Registration System (CRS). In the CRS each Danish resident is registered with a unique identification number together with information about the resident´s date of birth, sex, cohabitation status, migration etc. Each of the sampled individuals received an invitational letter explaining the purpose of the study. The invitation also contained a unique login for a secure webpage giving access to the questionnaire. Those without Internet access had the opportunity to complete the questionnaire by telephone interview. Recipients who had not responded within two weeks were sent a reminder letter. After another two weeks, a private telemarketing company contacted non-respondents. The data collection took place from June to December 2012.

### The questionnaire

The questionnaire was designed to assess the occurrence of 44 predefined specific and non-specific cancer symptoms as well as general and frequent symptoms. The symptoms of attention for this paper were pelvic pain, post-menopausal bleeding, bleeding during intercourse, and pain during intercourse. These symptoms were selected through an extensive literature search including national and international guidelines [3, 15–18]. The process of designing, pilot testing and field testing the questionnaire is described in detail elsewhere [19].

The respondents were asked whether they had experienced one or more of the predefined symptoms within the preceding four weeks. In addition to confirming or denying the presence of each symptom, the respondents had the possibility to reply, “Do not wish to answer”. An additional answering category "not relevant for me" was present for three of the symptoms; postmenopausal bleeding, pain during intercourse and bleeding during intercourse. Follow up questions for each reported symptom included the onset of the symptom (“Less than one month ago”, “1–3 months ago”, “3–6 months ago” or “more than six months ago”). Further, for each reported symptom the respondents were asked whether they had contacted the GP regarding the symptom. If the respondents answered that they had not contacted their GP about a given symptom, they were asked whether they had any of the four predefined considerations: “I would be too embarrassed”, “I would be worried about wasting the doctor’s time”, “I would be worried about what the doctor might find”, and “I was too busy to make time to go to the doctor”. Further, this item contained an open text box to allow the respondents to state other considerations, which were merged into a fifth category in this paper, “the other category”.

To explore management of symptoms with regard to possible contemporary barriers, only symptoms with onset up to six months prior to answering the questionnaire were included for this study.

### Register data

Using the individual identification numbers in the CRS, socioeconomic data were obtained from Statistics Denmark for each respondent. The variables of interest were highest obtained educational level, equivalence-weighted disposable income, employment status, marital status and ethnicity.

### Statistical analysis

The proportion of all eligible women, women with at least one recent-onset gynecological alarm symptom and women with at least one recent-onset gynecological alarm symptom that lead to GP contact are presented with the distribution of each covariate. Further, the proportion of women with recent-onset gynecological cancer alarm symptoms and no contact to the GP with these symptoms are presented as percentages for each symptom. Proportions of women reporting the five barriers for each symptom that did not lead to GP contact are presented as percentages. Women indicating that they did not wish to answer the questions were included, their answers treated as missing. For all proportions the confidence intervals were calculated using binomial distribution.

Logistic regression models were used to calculate unadjusted and adjusted odds ratios (ORs) for associations between each covariate and reported barrier. Covariates showing a significant association with each barrier in the crude model were considered possible confounders, and subsequently included in the adjusted models.

The covariates were categorized as follows: Age: 20–39, 40–59, or 60 + years old. Highest obtained educational level: low (< 10 years, i.e. primary/lower secondary school); middle (10–14 years, i.e. vocational or higher secondary school) or high (≥ 15 years, i.e. short-, medium- or long-term higher education). Equivalence-weighted disposable income: low (1st quartile), middle (2nd and 3rd quartile) or high (4th quartile) based on the entire household income after taxation, adjusted for number of persons in the household in the year of answering the questionnaire. Employment status: Working, pensioner, outside workforce or disability pension. Marital status: Married/cohabiting or single. Ethnicity: Danish origin or immigrants/descendants of immigrants.

Data analyses were conducted using STATA statistical software 13.1 (StataCorp, College Station, TX, USA). All tests used a significance level of *p* < 0.05.

## Results

**Fig. 1:**
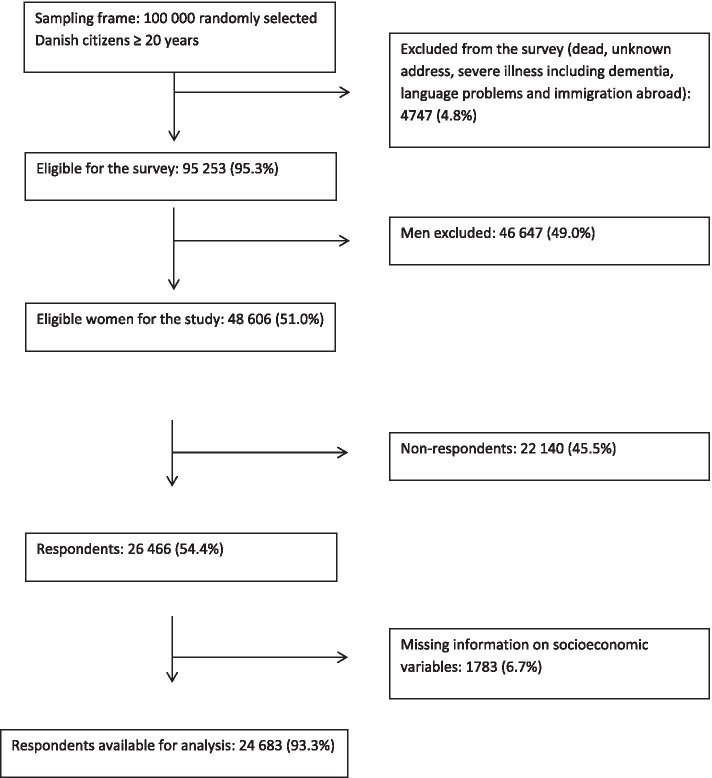
Study population

A total of 26 466 women participated in the survey, which comprises 54.4% of the eligible participants. Of these 24 683 had answered all relevant items and had no missing register data in Statistics Denmark (Fig. 1). Participants’ median age was 51 years (interquartile range (IQR) 39–63), compared to 53 years (IQR 37–71) for non-participants.

Between 1.25% (post-menopausal bleeding) and 2.49% (pain during intercourse) of the women indicated that they did not wish to answer the questions.

Some 2782 (11.3%) of the respondents reported at least one newly-onset gynecological alarm symptom. Of those, 2181 (78.4%) had not contacted their GP (Table 1).

**Table 1:** Socioeconomic characteristics for women reporting at least one newly onset gynecological alarm symptom

	All women	At least one newly onset symptom, N (%)	GP contact with at least one newly onset symptom, N (%)
**All**	24 683 (100.0%)	2782 (100.0%)	2181 (100.0%)
**Age**			
20–39	5554 (22.5%)	1275 (45.8%)	1036 (47.5%)
40–59	10 680 (43.3%)	1237 (44.5%)	967 (44.3%)
60 +	8449 (34.2%)	270 (9.7%)	178 (8.2%)
**Labour market affiliation**			
Working	16 429 (66.6%)	2269 (81.6%)	1797 (82.4%)
Pension	5820 (23.6%)	167 (6.0%)	117 (5.4%)
Out of workforce	1360 (5.5%)	228 (8.2%)	184 (8.4%)
Disability pension	1074 (4.4%)	118 (4.2%)	83 (3.8%)
**Equivalence weighted disposable income**			
Low (1^st^ quartile)	4208 (17.0%)	559 (20.1%)	436 (20.0%)
Middle (2^nd^ and 3^rd^ quartile)	13 055 (52.9%)	1543 (55.5%)	1223 (56.1%)
High (4^th^ quartile)	7420 (30.1%)	680 (24.4%)	522 (23.9%)
**Ethnicity**			
Danish	23 155 (93.8%)	2579 (92.7%)	2043 (93.7%)
Immigrants and descendants of immigrants	1528 (6.2%)	203 (7.3%)	138 (6.3%)
**Marital status**			
Single	6326 (25.6%)	686 (24.7%)	539 (24.7%)
Married/cohabiting	18 357 (74.4%)	2096 (75.3%)	1642 (75.3%)
**Educational level**			
Low (< 10 years)	3069 (12.4%)	214 (7.7%)	151 (6.9%)
Middle (10–14 years)	12 644 (51.2%)	1461 (52.5%)	1141 (52.3%)
High (≥ 15 years)	8970 (36.3%)	1107 (39.8%)	889 (40.8%)

The proportions of women without GP contact ranged from 64.6% (postmenopausal bleeding) to 78.1% (pain during intercourse) (Table 2).

**Table 2:** The proportion of women with GP contact regarding the symptoms

Symptom	Number of women	Women without GP contact regarding the symptom, N (%)
Pelvic pain	2071	1600 (77.3%)
Postmenopausal bleeding	181	117 (64.6%)
Pain during intercourse	787	615 (78.1%)
Bleeding during intercourse	297	220 (74.1%)
At least one symptom	2782	2181 (78.4%)

Between 32.3% (bleeding during intercourse) and 45.3% (postmenopausal bleeding) of the respondents reported no barriers for GP contact. The proportions of reported barriers ranged from 7.5% for being too embarrassed (pelvic pain) to 26.8% for being too busy (bleeding during intercourse). Overall, being too busy to go to the doctor was the most commonly reported predefined barrier, which was reported by between 20.2% (pelvic pain) and 26.8% (bleeding during intercourse). Between 26.2% (pain during intercourse) and 33.4% (pelvic pain) reported “other” barriers (Table 3).

**Table 3:** The proportion of reported barriers towards GP contact for each symptom, N (%)

	Pelvic pain	Postmenopausal bleeding	Bleeding during intercourse	Pain during intercourse
Being too embarrassed	120 (7.5%)	8 (6.8%)	38 (17.3%)	114 (18.5%)
Wasting the GP's time	275 (17.2%)	15 (12.8%)	39 (17.7%)	116 (18.9%)
Worried about what the GP might find	226 (14.1%)	11 (9.4%)	40 (18.2%)	107 (17.4%)
Being too busy	323 (20.2%)	25 (21.4%)	59 (26.8%)	137 (22.3%)
Other	535 (33.4%)	35 (29.9%)	67 (30.5%)	161 (26.2%)
None	539 (33.7%)	53 (45.3%)	71 (32.3%)	212 (34.5%)

Women aged 40–59 years had lower odds of reporting “being too embarrassed” (OR_adj_ 0.5, 95%-CI: 0.4–0.7) and reporting “worried about wasting the GP’s time” (OR_adj_ 0.7; 95%-CI 0.5–0.8) compared to the youngest age group. Further, women aged 60 + years of age had lower odds of reporting “worried about wasting the GP´s time”(OR_adj_ 0.5; 95%-CI 0.3–0.9) and lower odds of reporting “being too busy” (OR_adj_ 0.1, 95%-CI:0.1–0.3) compared to the youngest age group (Table 4).

**Table 4:** The association between the covariates and barriers for GP contact with at least one newly onset gynecological alarm symptom

	Being too embarrassed		Wasting the GP's time		Worried about what the GP might find		Being too busy		Other	
	OR; 95%-CI	OR_adj_; 95%-CI	OR; 95%-CI	OR_adj_; 95%-CI	OR; 95%-CI	OR_adj_; 95%-CI	OR; 95%-CI	OR_adj_; 95%-CI	OR; 95%-CI	OR_adj_; 95%-CI
**Age**										
20–39	1	1	1	1	1	1	1	1	1	1
40–59	**0.5; (0.4–0.7)**	**0.5; (0.4–0.7)**	**0.7; (0.5–0.8)**	**0.7; (0.5–0.8)**	0.8; (0.6–1.1)	0.8; (0.6–1.1)	0.9; (0.7–1.1)	1.0; (0.8–1.2)	1.0; (0.8–1.2)	1.1; (0.9–1.3)
60 +	0.8; (0.5–1.3)	0.6; (0.4–1.1)	**0.6; (0.4–0.9)**	**0.5; (0.3–0.9)**	1.1; (0.7–1.7)	1.0; (0.6–1.5)	**0.1; (0.1–0.3)**	**0.1; (0.1–0.3)**	0.8; (0.5–1.1)	1.1; (0.7–1.6)
**Labour market affiliation**										
Working	1	1	1	1	1	1	1	1	1	1
Pension	1.4; (0.8–2.5)	3.0; (0.8–11.3)	1.0; (0.6–1.6)	3.0; (0.9–9.5)	1.4; (0.9–2.4)	1.3; (0.5–3.3)	**0.1; (0.0–0.3)**	0.3; (0.1–1.3)	0.7; (0.4–1.0)	0.9; (0.4–1.9)
Out of workforce	1.5; (1.0–2.4)	1.3; (0.8–2.1)	**1.5; (1.0–2.1)**	1.4; (1.0–2.1)	**1.7; (1.1–2.4)**	**1.5; (1.0–2.3)**	0.9; (0.6–1.4)	0.8; (0.6–1.2)	**0.6; (0.4–0.8)**	0.7; (0.5–1.0)
Disability pension	1.2; (0.6–2.4)	1.3; (0.6–2.8)	1.1; (0.6–2.0)	1.3; (0.7–2.5)	0.9; (0.5–1.8)	0.8; (0.4–1.6)	**0.2; (0.1–0.5)**	**0.2; (0.1–0.5)**	**0.4; (0.2–0.7)**	0.6; (0.3–1.1)
**Equivalence weighted disposable income**										
Low (1^st^ quartile)	1	1	1	1	1	1	1	1	1	1
Middle (2^nd^ and 3^rd^ quartile)	**0.5; (0.4–0.8)**	**0.6; (0.4–0.8)**	**0.7; (0.5–0.9)**	**0.7; (0.5–0.9)**	0.8; (0.6–1.0)	0.9; (0.6–1.2)	0.8; (0.6–1.0)	0.9; (0.7–1.2)	**1.3; (1.0–1.7)**	1.2; (0.9–1.5)
High (4^th^ quartile)	**0.4; (0.2–0.5)**	**0.4; (0.2–0.7)**	**0.5; (0.4–0.7)**	**0.5; (0.4–0.8)**	0.7; (0.5–1.0)	0.9; (0.6–1.4)	0.9; (0.7–1.2)	1.1; (0.8–1.6)	**1.8; (1.4–2.4)**	**1.4; (1.0–2.0)**
**Ethnicity**										
Danish	1	1	1	1	1	1	1	1	1	1
Immigrants and descendants of immigrants	**1.7; (1.0–2.7)**	1.6; (1.0–2.6)	1.1; (0.7–1.7)	1.0; (0.7–1.6)	1.1; (0.7–1.8)	1.1; (0.7–1.8)	**2.0; (1.4–2.9)**	**2.1; (1.4–3.0)**	**0.5; (0.3–0.8)**	**0.5; (0.3–0.8)**
**Marital status**										
Single	1	1	1	1	1	1	1	1	1	1
Married/cohabiting	0.9; (0.7–1.3)	1.0; (0.7–1.4)	0.9; (0.7–1.2)	1.0; (0.7–1.2)	0.8; (0.6–1.0)	0.8; (0.6–1.1)	0.8; (0.6–1.0)	**0.8; (0.6–1.0)**	1.1; (0.9–1.3)	1.0; (0.8–1.3)
**Educational level**										
Low (< 10 years)	1	1	1	1	1	1	1	1	1	1
Middle (10–14 years)	0.8; (0.5–1.3)	0.7; (0.4–1.2)	0.8; (0.5–1.2)	0.7; (0.4–1.1)	0.8; (0.5–1.2)	0.8; (0.5–1.2)	**1.6; (1.0–2.6)**	1.2; (0.8–2.1)	**2.4; (1.5–3.9)**	**2.4; (1.5–4.0)**
High (≥ 15 years)	**0.6; (0.3–1.0)**	**0.5; (0.3–0.9)**	0.8; (0.5–1.3)	0.7; (0.4–1.1)	**0.6; (0.4–0.9)**	**0.6; (0.4–1.0)**	**1.7; (1.0–2.7)**	1.2; (0.7–2.0)	**4.5; (2.8–7.3)**	**4.6; (2.8–7.6)**

Bold denotes significant results (*p* < 0.05)

Women outside the workforce had higher odds of reporting “worried what the GP might find” (OR_adj_ 1.5, 95%-CI: 1.0–2.3) compared to those working, while women on disability pension had lower odds of reporting “being to busy” compared to those working (OR_adj_ 0.2, 95%-CI: 0.1–0.5) (Table 4).

Compared to those with a low income, women with middle and high income had lower odds of reporting “being too embarrassed” (OR_adj_ 0.6, 95%-CI: 0.4–0.8 and OR_adj_ 0.4, 95%-CI: 0.2–0.7, respectively) and “wasting the GP´s time” (OR_adj_ 0.7, 95%-CI: 0.5–0.9 and OR_adj_ 0.5, 95%-CI: 0.4–0.8, respectively) (Table 4).

Immigrants and descendants of immigrants had higher odds of reporting “being too busy” (OR_adj_ 2.1, 95%-CI: 1.4–3.0) compared to those of Danish origin (Table 4).

Married/cohabiting women had lower odds of reporting “being too busy” compared to single women (OR_adj_ 0.8, 95%-CI: 0.6–1.0) (Table 4).

Women with high educational level had lower odds of reporting “being too embarrassed” and “worried what the GP might find” (OR_adj_ 0.5, 95%-CI: 0.3–0.9 and OR_adj_ 0.6, 95%-CI 0.4–1.0, respectively) compared to those with low educational level. Women with middle and high educational level had higher odds of reporting “other” compared to those with low educational level (OR_adj_ 2.4, 95%-CI: 1.5–4.0 and OR_adj_ 4.6, 95%-CI: 2.8–7.6) (Table 4).

## Discussion

### Main findings

Less than 35% of women reporting gynecological cancer alarm symptoms contact the GP. Post-menopausal bleeding was the symptom with the highest proportion of GP contact, although nearly two third had not contacted their GP regarding postmenopausal bleeding. Bleeding during intercourse, only led to GP contact in 25.9.% of reported cases.

Women with intercourse-related symptoms had the highest proportions of reported barriers, whereas nearly half of those with postmenopausal bleeding did not report barriers. Being too busy was the most commonly reported predefined barrier overall.

In general, older women were less likely to report barriers towards GP contact with gynecological alarm symptoms. Women with higher income and educational level were less likely to be embarrassed and worried, but more often reported ‘other’ as a barrier. Individuals out of the workforce were more often worried, while women on disability pension had lower odds of reporting being too busy.

### Strengths and limitations of the study

A major strength of the study is its large number of randomly selected participants reflecting the general population.

The response rate of 54.4% is high compared to similar surveys [20]. Although the sample was randomly selected, some selection bias may remain. An overall responder analysis of the entire study cohort including both genders showed that respondents were more often cohabiting, had higher educational level, had higher income, were of Danish origin and more were affiliated with the workforce [21].

Another strength of the study is the use of high-quality data from national socioeconomic registers where detailed individual information is available, and the civil registration number enables accurate linkage.

The questionnaire design itself has some strengths and limitations. In order to explore contemporary management of symptoms, we only included symptoms experienced within the preceding four weeks and with onset six months prior to completion of the questionnaire. Although it may seem reasonable that individuals remember symptom experiences within this time frame, recall bias cannot be excluded. Additionally, respondents may have misunderstood questions, although several rounds of pilot testing were conducted to ensure correct interpretation of the items.

A strength of the questionnaire is the anonymity it provides. This may reduce information bias, particularly regarding sensitive topics such as pelvic symptoms.

The questionnaire being internet-based may have prevented older citizens from participating. Telephone interviews were provided to counter this and were primarily utilized by older participants. However, the telephone interviews may have reduced the respondents’ perceived privacy, leading to underreporting [19].

In addition to the predefined barriers, between 26.2% and 33.4% of the women report “other” barriers. Exploring the statements in the “other” category is however beyond the scope of this study, but the relatively high proportions of answers in this category indicate that other barriers than those predefined should be explored in future studies.

### Comparison with existing literature

In one study examining differences in barriers to presentation of a number of common cancer symptoms, Danish respondents were less likely to report being too embarrassed to present the symptom to their doctor with only 6% reporting this barrier [22]. We found that the proportion of respondents reporting being too embarrassed ranged from 6.8% to 18.5%. The differences are likely due to different symptoms assessed, as gynecological symptoms undoubtedly are of more intimate character. Further, our study addressed experienced symptoms rather than anticipated behavior, i.e. hypothetical situations, which may explain some of the differences. Other studies have also examined the role of psychological barriers in healthcare-seeking, [23–25] and found that worries about findings, followed by feeling too busy to go to the doctor, would be most likely to deter respondents from contacting their GP. We found that “being too busy” was the most common barrier, followed by “worrying about wasting the GP´s time”. This discrepancy may also be explained by differences in data collection.. We are not aware of other studies examining the relationship of each experienced barrier with each gynecological alarm symptom, though a British study found that fewer women anticipated contacting their GPs due to symptoms relating to intercourse, compared to post-menopausal bleeding or pelvic pain. Conversely to our findings, anticipated healthcare-seeking for pelvic pain and post-menopausal bleeding were almost equal [26].

Several studies have found socioeconomic gradients in cancer survival and advanced stage at diagnosis [27–29]. Our findings indicate that respondents with short educations and from low-income households are more likely to experience barriers toward help-seeking, possibly contributing to this issue. This is relatively consistent with findings from other studies [23, 24]. The study by Hvidberg et al. found that middle-income households reported fewest barriers, though, we found the lowest number of barriers among high-income households.

We found that ethnic minorities were more likely to report being too busy. This is in contrast with the findings of another Danish study, though their results pointed toward embarrassment, rather than busyness or worries about findings, as the most common barrier [24]. It is also comparable with a Canadian study finding that women of Indian origin were less likely to have pap smears done, compared to women of European origin [30]. This lower rate of healthcare seeking could not be explained solely by symptom awareness and may be partially due to higher psychological barriers. Only a small proportion of women in our study belonged to ethnic minorities, warranting the results to be interpreted with caution.

## Implications

Considering the role barriers play in diagnostic and treatment delay, knowledge about barriers towards GP contact is important to improve swift diagnosis. The high prevalence of the barriers involving either the women’s or the doctors’ time, may suggest low awareness of symptom significance among Danish women. It may also suggest worries about whether the doctor might think that the contact is unnecessary, worth noting in the context of doctor-patient relations.

The association of experiencing embarrassment or worries about wasting the GPs time with low socioeconomic status suggests more targeted and innovative initiatives to encourage GP visits, e.g. by means of social media accounts. These findings add to the increasing bank of knowledge pointing to the need for multifaceted interventions necessary for improving timely cancer diagnosis [31, 32].

## Conclusions

We found that the majority of women reported barriers towards GP contact with gynecological alarm symptoms. Being too busy to contact the GP was the most prevalent reported barrier overall. Younger age, lower educational level, lower income and being single was in general associated with higher odds of reporting barriers. Women with low income and low educational level were more likely to report embarrassment and worrying about the GP might find.

## Data Availability

The datasets generated and analyzed during the current study are not publicly available due to the data protection regulations of the Danish Data Protection, Statistics Denmark and the Danish Health and Medicines Authority. Further, ethical and legislative approval is conditional on publishing anonymized, summary data only. Access to data is strictly limited to the researchers who have obtained permission for data processing, and the dataset is still being used for ongoing research and publications. This permission was giving to the Research Unit of General Practice, Department of Public Health, University of Southern Denmark.

## Abbreviations

GP: General practitioner
OR: Odds ratio
CI: Confidence interval
